# Peak-MELD scores, a disease trajectory associated with reduced access to liver transplantation

**DOI:** 10.3389/fmed.2026.1761381

**Published:** 2026-06-26

**Authors:** Paul Jamme, Julian Allgeier, Dionysios Koliogiannis, Markus Guba, Christian M. Lange

**Affiliations:** 1 Department of Internal Medicine II, LMU University Hospital Munich, Munich, Germany; 2 Department of General, Visceral and Transplantation Surgery, LMU University Hospital Munich, Munich, Germany; 3 Department of Internal Medicine I, University Medical Center of the Johannes Gutenberg University Mainz, Mainz, Germany

**Keywords:** infection, liver transplantation, peak-MELD score, transplantation rate, waitlist mortality

## Abstract

The Model for End-Stage Liver Disease (MELD) score has been the primary allocation tool in the Eurotransplant region. However, certain patient groups remain disadvantaged. As diagnostic and therapeutic means evolve, regular revision of allocation criteria is necessary. Infectious diseases can lead to acute organ dysfunction and transient increases in MELD scores, whereas uncontrolled infections represent a contraindication to transplantation. In this retrospective study, we investigated peak-MELD scores, defined as a transient increase of at least five MELD points within 1 month, and evaluated their association with infections and liver transplantation outcomes. Among 109 patients listed for liver transplantation at LMU University Hospital between 2019 and 2022, 11% experienced a peak-MELD trajectory. It was associated with a significantly lower transplantation rate independent of baseline characteristics (HR = 0.37; 95% CI [0.15, 0.92]; *p* = 0.03). Patients with peak-MELD scores underwent more frequent waitlist status changes (median 2.50 vs. 0.00; *p* < 0.001), with infections representing the predominant cause of these changes (median 33.33% vs. 0.00%; *p* = 0.002). In this small single-center study, our findings suggest that a transient MELD increase associated with infectious complications may reflect a clinically vulnerable disease trajectory linked to reduced access to transplantation. Larger multicenter studies are needed to validate these findings and to further evaluate the implications of MELD fluctuations on waitlist outcome. At present, clinicians may consider dynamic disease courses when assessing liver transplantation urgency in patients with cirrhosis.

## Introduction

Liver transplantation (LT) is the only curative treatment option for patients with liver cirrhosis. Given the persistent shortage of donor organs, identifying patients at high risk of mortality is crucial (1). In the Eurotransplant region, the Model for End-Stage Liver Disease (MELD) score has served as the primary allocation tool. Based on laboratory parameters, it gives an objective estimation of 3-months mortality. Nevertheless, its predictive power is limited (2). In the context of acute decompensation, acute-on-chronic liver failure and hepatorenal syndrome, other liver-specific scores may be superior in mortality prediction (3–5). It is also widely recognized that certain patient groups, such as women, remain disadvantaged (6). Consequently, additional allocation criteria have been introduced to account for specific risk factors and alternative prioritization models are tested to improve equity for patients with acute-on-chronic liver failure (7). In the context of systemic inflammation and immune dysfunction, infectious diseases contribute to organ damage (8). Such infections may lead to increases in creatinine, bilirubin and international normalized ratio, thereby increasing MELD scores. However, uncontrolled infections represent a contraindication to LT and necessitate temporary removal from the active waitlist. In clinical practice, recovery from infections allows for reactivation on the waitlist. On the other hand, improvement of laboratory parameters often results in a decline in the MELD score. It therefore remains unclear, whether a return to baseline MELD values truly reflects clinical stabilization or whether infection-associated MELD fluctuations contribute to reduced access to LT. The present study explores MELD score dynamics and highlights implications of transient peak-MELD scores associated with acute infections.

## Materials and methods

We retrospectively analyzed 109 patients newly introduced to the LT waitlist at the LMU Hospital between 2019 and 2022. All patients had liver cirrhosis and were allocated through lab MELD scores, while those with acute liver failure, combined organ transplantation or exception criteria were excluded. Patient management followed the local standard operating procedures as well as national guidelines on liver cirrhosis. MELD scores were recalculated either at predefined intervals or when a higher score became apparent following the German guideline for organ transplantation. This guideline illustrates the clinical relevance of MELD score categories by presenting the mortality risks associated with MELD values of 10, 15, 20 and 30 points. In this context, an increase by 5 MELD points reflects a marked increase in short-term mortality. Acute complications frequently cause rapid surges in MELD score, whereas a gradual increase over longer periods commonly reflects progressive deterioration of liver function. In order to capture relevant acute clinical deterioration, we defined a peak-MELD trajectory as an increase of ≥5 points within 1 month followed by a decrease in accordance with the Eurotransplant recertification schedule. Patients with and without a peak-MELD score were compared regarding clinical parameters, transplantation rate and mortality. Statistical analyses were performed using GraphPad Prism (Version 10.1.1; 2023) and R (Version 4.2.1; 2022). Normality of continuous variables was assessed using the Shapiro–Wilk test. Continuous variables were analyzed using unpaired *t*-tests or Mann-Whitney U tests, while categorical variables were compared using Fisher’s exact test. Time-to-event outcomes were compared using the Log–Rank test. Hazard ratios (HRs) with 95% confidence intervals (CIs) were estimated using Cox proportional hazards models. For non-normally distributed continuous variables, effect sizes were reported as Hodges–Lehmann differences with corresponding 95% CIs. The study was approved by the LMU University Hospital Ethics Committee (No. 24-1037) and all research was conducted in accordance with both the Declaration of Helsinki and the Declaration of Istanbul.

## Results

**FIGURE 1:**
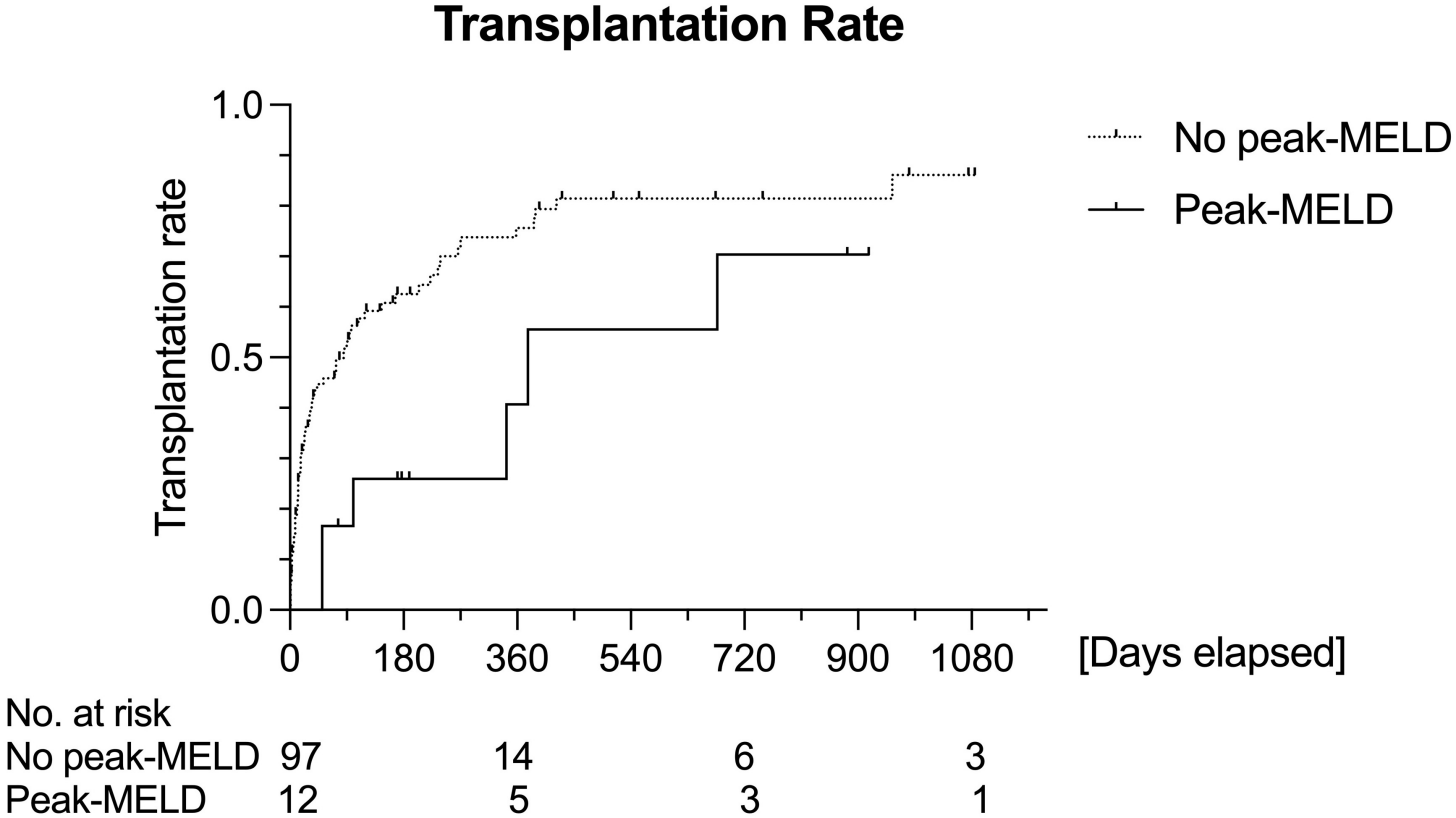
Kaplan-Meier analysis of time to liver transplantation in patients with and without a peak-MELD score. Patients who developed a peak-MELD score showed a significantly lower transplantation rate (Log–Rank test, χ^2^ = 4.01; *p* = 0.045) and a longer median time to transplantation (377 vs. 85 days).

**FIGURE 2:**
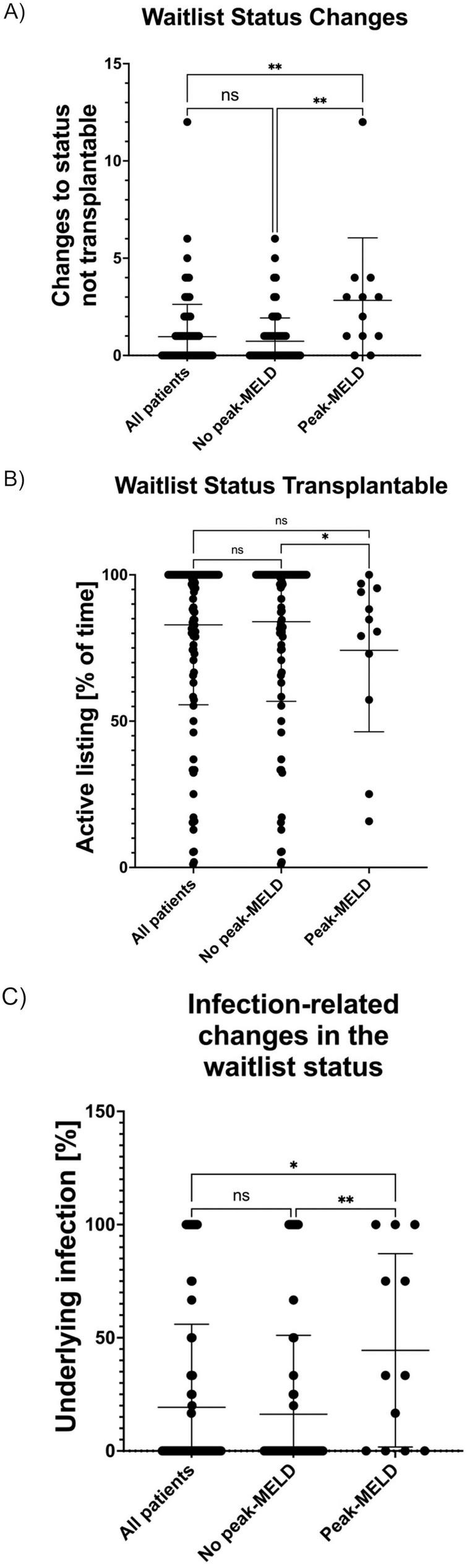
**(A)** Comparison of the frequency of changes in waitlist status between patients with and without a peak-MELD score. The diagram shows the absolute number of transitions to a “not transplantable” status. Patients with a peak-MELD score experienced significantly more changes compared to those without a peak-MELD score (median 2.50 vs. 0.00; Hodges–Lehmann difference 1.00; 95% CI [1.00, 3.00]; Mann–Whitney U test, *p* < 0.001). **(B)** Comparison of the time spent in waitlist status “transplantable.” The diagram shows the proportion of active listing time relative to total waitlist time. Patients with a peak-MELD score spent significantly less time in transplantable status (median 82.66% vs. 100.00%; Hodges–Lehmann difference –7.26%; 95% CI [–19.41%, –1.15%]; Mann–Whitney U test, **p ≤ 0.01). **(C)** Comparison of infection-driven waitlist status changes. The diagram shows the proportion of changes attributable to infections. Patients with a peak-MELD score experienced a significantly higher infection-associated change rate (median 33.33% of status changes due to infections vs. 0.00%; Hodges–Lehmann difference 16.67%; 95% CI [0.00%, 75.00%]; Mann–Whitney U test, *p* = 0.002). *Indicates *p* < 0.05.

**FIGURE 3:**
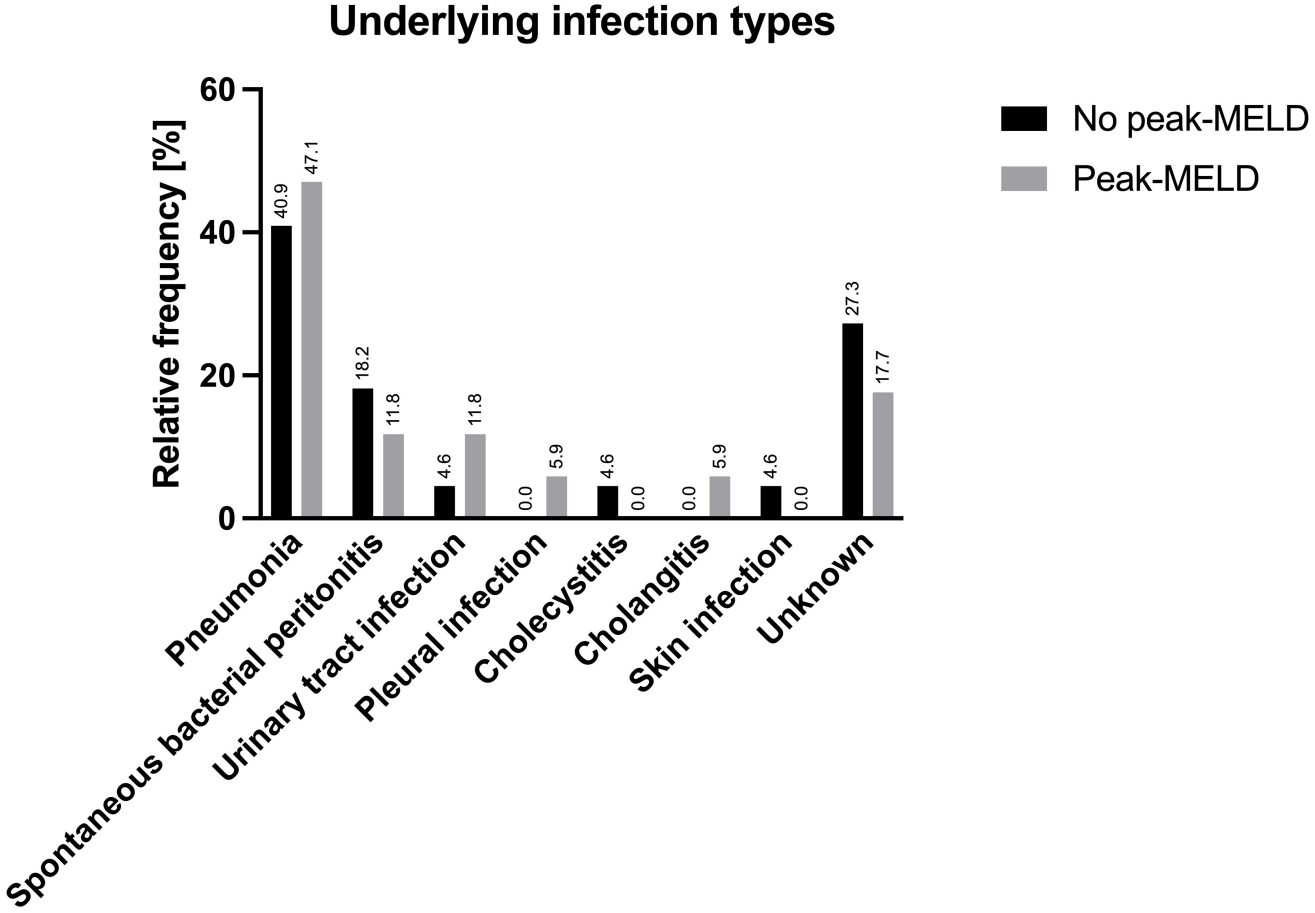
Underlying infection types for patients with and without a peak-MELD score. The absolute number of waitlist status changes due to infections was 17 and 22, respectively. The diagram shows the relative frequency of infections, expressed as percentages within each group. Comparisons between groups were performed using a two-sided Fisher’s exact test after grouping infections into pneumonia and non-pneumonia categories. No significant differences were observed (Fisher’s exact test, *p* = 0.66).

The mean follow-up time was 525 ± 453 days. Among the 109 patients, 70 (64.2%) underwent LT. The peak-MELD phenomenon occurred in 12 patients (11%). Baseline characteristics, including age, sex, blood type, Child-Pugh score, initial MELD and reMELD-Na score, body mass index, comorbidities, etiology of cirrhosis and laboratory parameters, did not significantly differ between the two groups (Table 1). However, patients who experienced a peak-MELD score showed a significantly lower transplantation rate (Log–Rank test, χ^2^ = 4.01; *p* = 0.045) and a longer median waiting time for transplantation (377 vs. 85 days; Figure 1). This result remained significant after adjustment for age, sex, initial MELD score and blood type (Cox proportional hazards model: HR = 0.37; 95% CI [0.15, 0.92]; *p* = 0.03). Additional variables independently associated with LT in the multivariable model included age (HR = 1.03; 95% CI [1.00, 1.06]; *p* = 0.04), initial MELD score (HR = 1.17; 95% CI [1.13, 1.22]; *p* < 0.0001) and blood type AB (HR = 9.77 compared to blood type O; 95% CI [2.29, 41.65]; *p* = 0.002). While waitlist mortality did not differ significantly between groups, patients with a peak-MELD score exhibited more frequent changes in waitlist status (median 2.50 vs. 0.00; Hodges–Lehmann difference 1.00; 95% CI [1.00, 3.00]; Mann–Whitney U test, *p* < 0.001; Figure 2A) and shorter durations in active transplantable status (median 82.66% vs. 100.00%; Hodges–Lehmann difference −7.26%; 95% CI [−19.41%, −1.15%]; Mann–Whitney U test, *p* = 0.01; Figure 2B). Notably, infections substantially contributed to the short active listing (median 33.33% of status changes due to infections vs. 0.00%; Hodges–Lehmann difference 16.67%; 95% CI [0.00%, 75.00%]; Mann–Whitney U test, *p* = 0.002; Figure 2C). Following a peak-MELD score, a markedly increased proportion of waitlist status changes was attributable to clinically relevant infections (median 75.00% vs. 0.00% overall; Hodges–Lehmann difference 50%; 95% CI [0.00%, 100.00%]; Mann–Whitney U test, *p* = 0.02). Among the most frequent infections in patients with and without peak-MELD trajectories were pneumonia (47.1% vs. 40.9%), spontaneous bacterial peritonitis (11.8% vs. 18.2%) and urinary tract infection (11.8% vs. 4.6%; Figure 3). These findings highlight the significance of a clinical disease course characterized by a sudden rise in the MELD score, frequent waitlist status changes, a high rate of infections and a reduced likelihood of LT.

**TABLE 1:** Baseline characteristics.

Baseline characteristics	All patients (*n* = 109)	No peak-MELD score (*n* = 97)	Peak-MELD score (*n* = 12)	*P*-value
Age [years]	54 (48.5–60.5)	55 (49–61)	50.5 (41.5–54.75)	0.06
Female gender [*n*, %]	34 (31.2%)	31 (32%)	3 (25%)	0.75
Blood type [n, %]				
Blood type O	37 (33.9%)	31 (32%)	6 (50%)	–
Blood type A	48 (44%)	46 (47.4%)	2 (16.7%)	–
Blood type AB	4 (3.7%)	4 (4.1%)	0 (0%)	–
Blood type B	20 (18.3%)	16 (16.5%)	4 (33.3%)	–
Child-score [n, %]				
Child-score A	4 (3.7%)	4 (4.1%)	0 (0%)	–
Child-score B	46 (42.2%)	42 (43.3%)	4 (33.3%)	–
Child-score C	59 (54.1%)	51 (52.6%)	8 (66.7%)	–
Initial MELD score [points]	20 (16–27.5)	21 (16–28.5)	18.5 (15.5–21)	0.22
Initial reMELD-Na score [points]	19 (16.5–26)	20 (16–27)	18.5 (16.8–20.3)	0.32
Body mass index [kg/m^2^]	26.9 ± 4.9	26.9 ± 4.9	27 ± 4.7	0.95
Arterial hypertension [*n*, %]	29 (26.6%)	23 (23.7%)	6 (50%)	0.08
Diabetes mellitus [*n*, %]	38 (34.9%)	35 (36.1%)	3 (25%)	0.54
Chronic kidney disease [*n*, %]	27 (24.8%)	23 (23.7%)	4 (33.3%)	0.49
Causes of liver cirrhosis [*n*, %]				0.96
Alcohol	54 (49.5%)	49 (50.5%)	5 (41.7%)	
Viral hepatitis	4 (3.7%)	3 (3.1%)	1 (8.3%)	
MASLD	7 (6.4%)	6 (6.2%)	1 (8.3%)	
Multifactorial	13 (11.9%)	11 (11.3%)	2 (16.7%)	
Other	31 (28.4%)	28 (28.9%)	3 (25%)	
Leukocyte count [G/L]	6.2 (3.8–7.9)	6.1 (3.8–8)	6.6 (4.9–7.1)	0.89
Platelet count [G/L]	79 (51–111)	82 (51–110)	63 (36.3–132)	0.65
Hemoglobin [g/dL]	8.9 (7.7–11)	8.9 (7.8–11)	9.1 (7.5–12)	0.91
Sodium [mmol/L]	135.9 ± 5.5	136 ± 5.8	135.2 ± 2.8	0.66
Creatinine [mg/dL]	1.2 (0.8–1.6)	1.2 (0.88–1.7)	0.8 (0.7–1.5)	0.11
Albumin [g/dL]	3.3 ± 0.6	3.3 ± 0.5	3.1 ± 0.7	0.37
Bilirubin [mg/dL]	5.5 (2.8–12)	5.5 (2.7–12)	4.1 (3.2–6.4)	0.5
INR []	1.7 (1.4–2.1)	1.7 (1.4–2.1)	1.6 (1.5–1.8)	0.5

Data are presented as mean ± standard deviation, median (interquartile range: p25–p75) or counts (percentages), as appropriate. *P*-values were calculated between patients with and without a peak-MELD score using unpaired *t*-tests and Mann–Whitney U tests (continuous variables) or Fisher’s exact tests (categorical variables). MASLD, metabolic dysfunction-associated steatotic liver disease; INR, international normalized ratio.

## Discussion

In our study, we describe peak-MELD scores as transient events associated with reduced transplantation rates in patients awaiting LT. Moreover, peak-MELD scores were associated with frequent infectious complications, while the distribution of infection types did not differ significantly between groups. These findings are consistent with previous studies demonstrating the increased susceptibility of patients with liver cirrhosis to infectious complications (9). Although infections may transiently increase MELD scores through acute organ dysfunction, they simultaneously render patients temporarily ineligible for LT. While the clinical relevance of peak-MELD scores remains to be established, this dynamic disease trajectory may indicate unintended disadvantages within allocation systems based on static scores. In the Eurotransplant region, exception criteria were introduced to compensate for the increased mortality of specific diseases, for example hepatocellular carcinoma. Since March 2025, the reMELD-Na score is used to do justice to the high mortality resulting from hyponatremia. As the allocation system continues to develop, we want to stimulate discussion on dynamic disease trajectories and potential strategies to overcome disadvantages associated with MELD fluctuations. Future allocation models may incorporate corrective mechanisms that account for transient clinical deterioration. For example, a peak-MELD score could trigger a temporary stabilization of the MELD score, that could help maintain access to LT. In this regard, existing static scores and re-defined dynamic approaches could complement each other and improve waitlist equity. Nevertheless, the feasibility and potential unintended consequences require careful evaluation. Our study has limitations. It is limited by its retrospective design and single-center cohort. The peak-MELD group only comprises 12 individuals, which reduces statistical power. With 70 transplantations and 5 variables, the cox proportional hazards model may be underpowered and prone to overfitting. Our study remains observational as we cannot state causality between peak-MELD trajectories and reduced transplantation rates. Other acute complications, such as hepatic encephalopathy, esophageal variceal bleeding or acute kidney injury, may likewise serve as markers of disease severity and be associated with fluctuations in MELD scores. Our findings should therefore be interpreted as hypothesis-generating. They describe MELD fluctuations as a clinical phenomenon and underscore the need for further investigation of dynamic MELD trajectories in patients listed for liver transplantation.

## Data Availability

The original contributions presented in this study are included in this article/supplementary material, further inquiries can be directed to the corresponding author.
